# Impact of neighbourhood-level socioeconomic status, traditional coronary risk factors, and ancestry on age at myocardial infarction onset: A population-based register study

**DOI:** 10.1186/s12872-022-02880-7

**Published:** 2022-10-26

**Authors:** Mathias Øie Kolden, Ståle H. Nymo, Erik Øie

**Affiliations:** Department of Internal Medicine, Diakonhjemmet Hospital, Oslo, Norway

**Keywords:** Socioeconomic status (SES), Socioeconomic disadvantage, Acute myocardial infarction (AMI), Non-ST-elevation myocardial infarction (NSTEMI), Coronary risk factors, Ancestry, Ethnicity

## Abstract

**Background:**

There is consensus that low socioeconomic status (SES) is associated with an increased risk of acute myocardial infarction (AMI), but the extent to which traditional coronary risk factors and other characteristics of low SES mediate this effect remains uncertain. This study examined AMI patients residing in neighbouring city districts with the same local hospital despite having among the most considerable differences in mean SES in Norway. Our purpose was to assess low SES as a coronary risk factor and examine whether traditional coronary risk factors or ancestry mediate this effect.

**Methods:**

Six hundred six patients (215 and 391 with a low and high neighbourhood-level SES, respectively) admitted to Diakonhjemmet Hospital with non-ST-elevation myocardial infarction (NSTEMI) between 2014 and 2017, entered analysis. Data from the Norwegian Myocardial Infarction Register were used to identify patient characteristics, and the STATA/SE 15.1 software was used to perform the statistical analyses.

**Results:**

Patients from socioeconomically disadvantaged city-districts had a 4.9 years earlier onset of AMI (68.99 vs. 73.89 years; p < 0.001) and a higher prevalence of previous AMI, known diabetes, and current smokers (36% vs. 27%, 25% vs. 12%, and 33% vs. 17%, respectively; all p ≤ 0.05). When only comparing patients with a first time AMI, an even greater difference in the age at AMI onset was found (6.1 yrs; p < 0.001). The difference in age at AMI onset remained statistically significant when adjusting for traditional coronary risk factors (3.28 yrs; 95% confidence interval (CI) 1.11−5.44; p = 0.003), but not when adjusting for presumed non-Northwest-European ancestry (1.81 yrs; 95% CI −0.55 to 4.17; p = 0.132).

**Conclusion:**

This study supports earlier research showing an increased risk of AMI in socioeconomically disadvantaged individuals. In our population, presumed non-Northwest-European ancestry could entirely explain the increased risk, whereas traditional coronary risk factors could only partly explain the increased risk.

## Introduction

Cardiovascular disease has for many decades been the leading cause of morbidity and mortality globally [1], and atherosclerosis is the underlying predominant cause [2]. Fortunately, due to healthier behaviours, especially reduced cigarette smoking, and improved acute and prophylactic treatment, both incidence and mortality of coronary heart disease (CHD) have dropped dramatically during the last four to five decades in high-income countries [3, 4].

However, not all population groups in these countries have experienced the same degree of reduction in the incidence of acute myocardial infarction (AMI) [4]. Previous studies have shown that low socioeconomic status (SES) is associated with a higher incidence and mortality of AMI and other health outcomes [5–12]. Several possible mechanisms mediating the effect of low SES on CHD have been investigated. There is consensus that traditional coronary risk factors mediate a considerable part of this effect, but that individual characteristics of low SES may also contribute [10, 13, 14]. Psychological, work-related, and neighbourhood-related risk factors, as well as risk factors in women during pregnancy, risk factors in childhood, and inequalities in access to health care may be of importance [15]. Race/ethnicity, which in this paper will be referred to as ancestry, capturing the common geographical origin, language, culture, genetic ancestry, and social history of particular groups, has also been shown to act in a complex interaction with SES and be an independent risk factor for CHD [16, 17]. Therefore, variations in ancestry across socioeconomic groups might mediate part of the effect of low SES on CHD. However, how traditional coronary risk factors, ancestry and individual characteristics of SES combine to affect socioeconomic inequalities in CHD have yet to be entirely understood.

To investigate the role of SES as a coronary risk factor, we aimed to compare AMI patients from a region where the population has a mean low SES with AMI patients from a region where the population has a mean high SES. To minimise potential bias, our study population was included from two regions of the same city with proximity to each other and having the same local hospital. Our main purpose was to investigate whether individuals with a low SES (exposure) have an earlier onset of AMI (outcome) and a higher prevalence of traditional coronary risk factors (outcome) than individuals with a high SES. If a difference could be found, our aim was to further investigate whether ancestry is a mediator of this effect (confounding factor).

## Methods

### Study population

We collected data registered in the Norwegian Myocardial Infarction Register for all patients admitted to Diakonhjemmet Hospital with non-ST elevation myocardial infarction (NSTEMI) in 2014–2017 (n = 840). Diakonhjemmet Hospital is the local hospital for the three western districts of Oslo (Frogner, Vestre Aker, and Ullern), with 144,000 inhabitants combined. The hospital has since 2013 also served as one of two primary local hospitals for patients residing in the three north-eastern districts of Oslo (Stovner, Grorud, and Alna) who present with acute medical conditions. The north-eastern districts have 111,000 inhabitants combined. Although there is a very short geographical distance between the western and north-eastern regions (less than 7 km), there is a large difference in income, highest completed education, cramped living conditions, and the number of social assistance recipients between the two populations (Table 1) [18–24]. Low SES is often defined as low household income or educational level less than high school. As shown in Table 1, approximately $1/5$ of the population in the north-eastern districts of Oslo have an income below 60% of the national median income and $1/3$ of the population have high school as the highest completed education. There is also a considerable difference in the number of first- and second-generation immigrants between the two populations [25].

**Table 1:** Indicators of socioeconomic status (SES) and immigration in the western and north-eastern districts of Oslo

		Western districts of Oslo			North-eastern districts of Oslo		
		Vestre Aker	Ullern	Frogner	Alna	Grorud	Stovner
Income	Average income (NOK^a^) 2019^b^	833 000	752 000	668 000	405 000	395 000	385 000
	Low income (%) 2017^c^	9.4	8.3	15.9	18.4	18.9	22
Highest completed education	University or college (%) 2017	63.1	62.5	61.9	31.1	29.4	24.7
	Vocational school (%) 2017	2.5	2.7	2.4	2.1	2	1.7
	High School (%) 2017	21.9	23.1	23.1	33.9	34	35
	Primary school (%) 2017	12.6	11.7	12.6	32.9	34.6	38.7
Socioeconomic status^d^		0.77	0.73	0.63	0.39	0.38	0.35
Social assistance recipients (%) 2016		1.7	1.5	3.1	3.1	5.3	4.9
Overcrowded individuals (%) 2019^e^		8.2	8.7	16.1	29.3	27.4	26.8
Life expectancy at birth	Men (years) 2013–2017	83.4	82.4	81.8	79.3	77.4	80.2
	Women (years) 2013–2017	87.2	85.4	84.5	83.2	81.4	83
Share of 1st and 2nd generation immigrants	Other than East-Europe (%) 2018^f^	6.6	7.7	10.3	40.4	38.4	46.4
	East-Europe (%) 2018	4.7	5.2	6.3	10.2	8.8	7.6

^a^1 NOK ~ 0.10€/0.12$. ^b^Average annual gross income per inhabitant > 16 years old. ^c^Percentage of inhabitants in private households with annual net income per consumption unit 60% below median income (EU-scale) excluding households consisting of students and children < 18 years old living alone. ^d^A constructed formative measurement where the variables education, income, and employment are converted to the same scale, and the average of these variables are equally weighted. ^e^The number of rooms in the household is less than the number of people, or one person lives in one room and the number of square meters (p-areal) is less than 25 m^2^ per person. ^f^Immigrants from Asia, Africa, South- and Central-America, and Turkey

STEMI patients are not included in this study as they are initially treated at Oslo University Hospital, which has invasive cardiac service, and since the outpatient follow-up of the patients from the north-eastern districts of Oslo is localised at Akershus University Hospital, most of these patients are transferred from Oslo University Hospital to Akershus University Hospital rather than Diakonhjemmet Hospital.

**Fig. 1:**
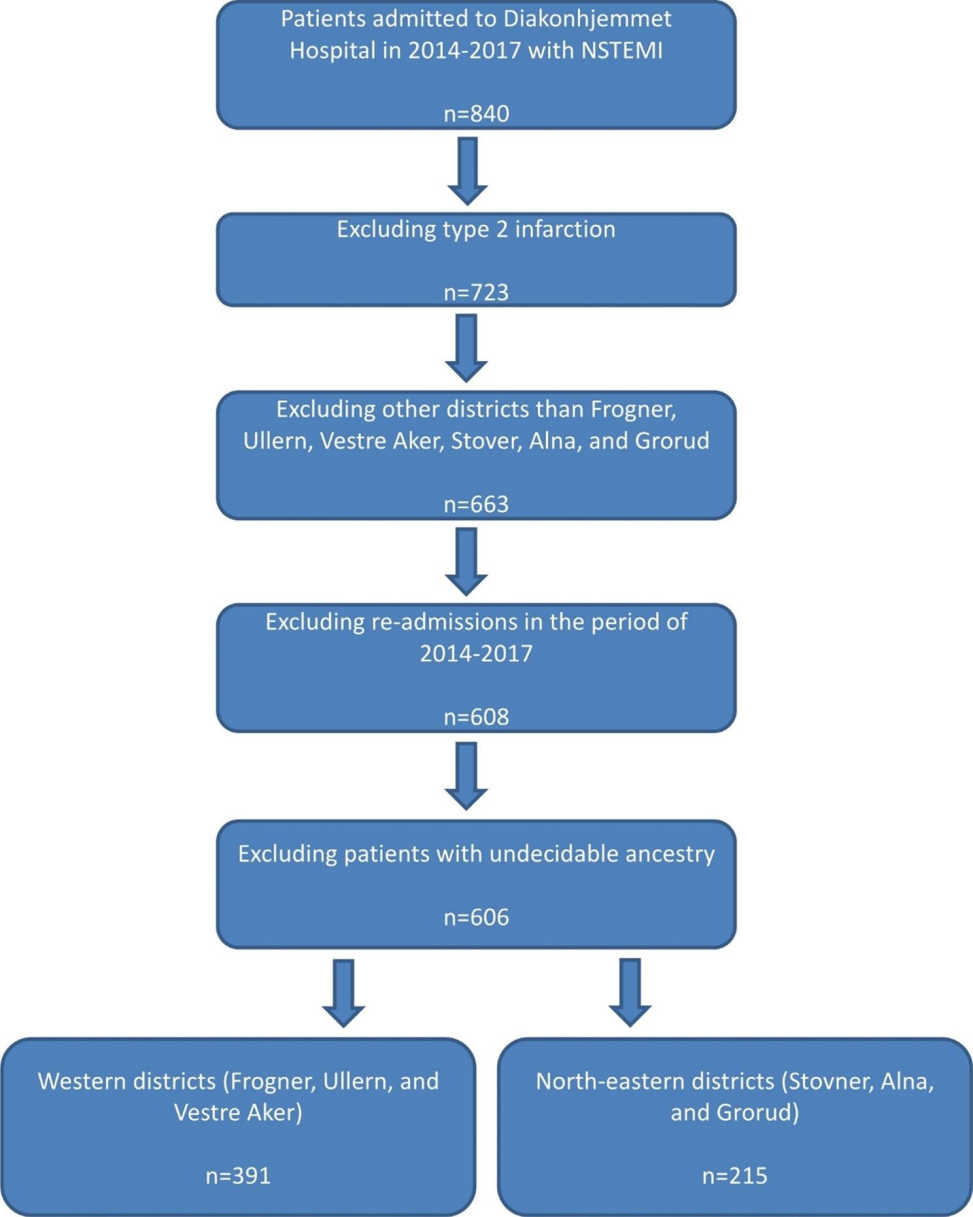
Flow chart for inclusion and exclusion of patients to the study

Patients with type 2 myocardial infarction were excluded (n = 117) since we aimed to investigate the risk for CHD. Furthermore, using the zip codes registered for the AMI patients admitted to Diakonhjemmet Hospital, patients from districts other than Frogner, Vestre Aker, Ullern, Stovner, Grorud, and Alna were excluded (n = 60). Re-admissions in the period (n = 55) and patients with undecidable ancestry (n = 2) were excluded, and we were left with 606 patients, which was 26% of all cases of NSTEMI in Oslo during the study period. There were 391 patients from the three western districts (high SES group) and 215 patients from the three north-eastern districts (low SES group) (Fig. 1). The total number of patients with NSTEMI (type 1 infarction) from the north-eastern districts of Oslo in the study period was 524 patients, of which 215 (41%) of these patients were admitted to Diakonhjemmet Hospital for treatment.

There are far more individuals with non-western ancestry in the north-eastern districts of Oslo than in the western ones (Table 1). In the north-eastern districts of Oslo, 38.4–46.4% are immigrants from Asia, Africa, South- and Central-America, and Turkey, whereas 7.6–10.2% are immigrants from East-Europe. Ancestry is not registered in the Norwegian Myocardial Infarction Register. However, we had available social security numbers for all patients, and using the electronic medical record system at Diakonhjemmet Hospital, we identified the names of the included patients. Based on the names and the information in the patient journals, patients were grouped according to whether or not they had presumed Northwest-European ancestry. In the low SES group, we found that 59 patients (27.4%) had presumed ancestry from elsewhere than Northwestern Europe. Corresponding numbers in the high SES group were 16 patients (4.1%). Of the 531 individuals with presumed Northwest-European ancestry, only 11 individuals had presumed ancestry from outside Scandinavia.

### Study outcomes

The primary endpoint of this study was the age at onset of AMI when comparing the low and high SES group. Secondary endpoints were the prevalence of previous AMI and the prevalence of traditional coronary risk factors when comparing the low and high SES group. If a difference in the age at AMI onset between the low and high SES group was found, a prespecified secondary endpoint was the extent to which ancestry confounds this relation.

Previous AMI was defined regardless of infarction type and ECG diagnosis, prior diagnosis of diabetes was defined as known diagnosis with diabetes mellitus type 1 or 2, prior diagnosis of hypertension was defined as prior or ongoing treatment for hypertension, and cigarette smoking was defined as patients that had been smoking the last month. Body mass index (BMI) and serum levels of low-density lipoprotein (LDL) cholesterol were measured at hospital admission.

### Statistical analyses

We used the STATA/SE 15.1 software to perform the statistical analyses. Frequency table and Pearson’s chi-square test were used to assess differences in the categorical variables (cigarette smoking, diabetes, hypertension, and previous AMI) between the low and high SES group. Similarly, Student’s *t*-test was used to assess differences in the averages of the continuous variables (age at AMI onset, BMI, and level of LDL cholesterol) between the low and high SES group. For the primary endpoint, age at AMI onset, we performed corresponding analyses when only including patients admitted with a first time AMI. For LDL-cholesterol and BMI, corresponding analyses were performed when patients using statins and stated active smoking, respectively, at the time of admission were excluded to adjust LDL-cholesterol for the use of statins and BMI for cigarette smoking status as nicotine may be an appetite suppressant. Since a difference in the primary endpoint was found, all analyses were repeated excluding patients with a presumed non-Northwest-European ancestry. To further explore ancestry as a possible confounder, we conducted multiple regression analyses with patient age as the dependent variable (outcome) and SES group (exposure), traditional coronary risk factors (possible confounder) and ancestry (possible confounder) as the independent variables. Multiple imputation was used to address missing values (Table 2). For all analyses, p-value ≤ 0.05 was considered statistically significant.

**Table 2:** Number of individuals with missing data for all variables in the western (mean high SES) and north-eastern (mean low SES) districts of Oslo *BMI* Body mass index

	Total population (n = 606)		Presumed Northwest-European ancestry (n = 531)	
	Western districts (n = 391)	North-eastern districts (n = 215)	Western districts (n = 375)	North-eastern districts (n = 156)
Smoking status	73	27	69	22
Hypertension	29	1	27	1
Diabetes mellitus	23	2	22	2
LDL-cholesterol	180	98	173	75
BMI	151	100	143	66
Use of statins	23	2	22	2
Previous AMI	30	4	29	4

## Results

### Patient age

As shown in Table 3, the mean age of the patients admitted with AMI was 4.9 years lower (68.99 vs. 73.89 yrs) in the low SES group compared with patients from the high SES group (p < 0.001). When patients with presumed non-Northwest-European ancestry were excluded, there was no significant difference in mean age at AMI onset between the low SES and high SES group (p = 0.223). When only including patients with no previous AMI, the difference in mean age at AMI onset between the low and high SES group increased to 6.1 years (65.88 vs. 71.97 yrs; p < 0.001). As before, there was no significant difference in mean age at AMI onset between the low SES and high SES group when patients with presumed non-Northwest-European ancestry were excluded (p = 0.195).

**Table 3:** Mean age at AMI onset and percentage of previous AMI in the western (mean high SES) and north-eastern (mean low SES) districts of Oslo

	Total population		Presumed Northwest-European ancestry	
	Western districts	North-eastern districts	Western districts	North-eastern districts
Mean age (years) at AMI onset	73.89	68.99**	74.36	72.78
Mean age (years) at AMI onset when only including patients with a first time AMI	71.97	65.88**	70.27	72.49
Previous AMI (%)	27.42	35.55*	27.17	38.16*

*p ≤ 0.05 and **p ≤ 0.001 compared with the corresponding population in the western districts. The number of patients reported having a first time AMI were 262, 136, 252, and 94 in the four populations, respectively. Previous myocardial infarction was reported as unknown in 30, 4, 29, and 4 patients in the four populations, respectively

### Previous AMI

In the low SES group, significantly more patients had previously had AMI compared with patients from the high SES group (36% vs. 27%; p = 0.042), also after excluding patients with presumed non-Northwest-European ancestry (38% vs. 27%; p = 0.014) (Table 3).

### Occurrence of traditional coronary risk factors

#### Cigarette smoking

**Fig. 2:**
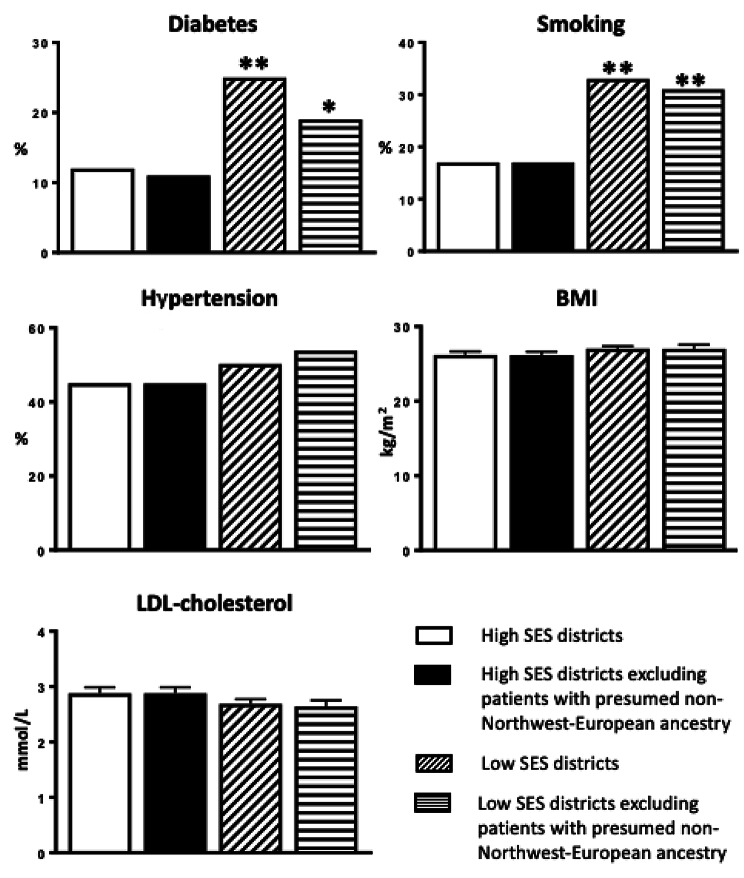
Distribution of traditional coronary risk factors. BMI is not adjusted for cigarette smoking, and LDL-cholesterol is not adjusted for the use of statins. See Table 3 for missing data. *p ≤ 0.05 and **p ≤ 0.001 compared with the corresponding population in the western district *BMI* Body mass index

In the low SES group, 1.94-fold as many were smokers compared with patients from the high SES group (33% vs. 17%; p < 0.001). When patients with presumed non-Northwest-European ancestry were excluded, 1.82-fold more individuals smoked in the low SES group (31% vs. 17%; p = 0.001) (Fig. 2).

#### Diabetes mellitus

In the low SES group, 2.08-fold as many had known diabetes compared with patients from the high SES group (25% vs. 12%; p < 0.001). When patients with presumed non-Northwest-European ancestry were excluded, 1.73-fold more individuals had diabetes in the low SES group (19% vs. 11%; p = 0.018) (Fig. 2).

#### LDL-cholesterol

The mean value of LDL cholesterol did not differ significantly between the low SES and high SES group (p = 0.157) regardless of whether patients with presumed ancestry from elsewhere than Northwestern Europe were excluded (p = 0.088) or not (Fig. 2). When only evaluating patients who did not receive treatment with statins at the time of admission, the result was still not significant (p = 0.152). However, when also excluding patients with presumed non-Northwest-European ancestry, the mean value was 3.23 mmol/L in the high SES group compared with 2.82 mmol/L in the low SES group (p = 0.013).

#### Obesity

The mean value for BMI was not significantly different between the low SES and high SES group (p = 0.143) regardless of whether patients with presumed ancestry from elsewhere than Northwestern Europe were excluded (p = 0.192) or not (Fig. 2). When only evaluating patients who did not smoke at the time of admission, there were still no significant differences regardless of whether patients with presumed ancestry from elsewhere than Northwestern Europe were excluded (p = 0.132) or not (p = 0.143).

#### Hypertension

There was no significant difference in the number of patients who had previously received or who were on treatment for hypertension between the low SES and high SES group (p = 0.206) regardless of whether patients with presumed ancestry from elsewhere than Northwestern Europe were excluded or not (p = 0.060) (Fig. 2).

### Underlying risk factors

#### Traditional coronary risk factors

When adjusting for traditional coronary risk factors such as cigarette smoking status, known diabetes, known hypertension, BMI, and LDL cholesterol, also including statin use, the difference in age at AMI onset between the low SES and high SES group was still statistically significant (3.28 yrs; 95% confidence interval (CI) 1.11−5.45; p = 0.003) (Table 4). Smoking was associated with a 7.22 years earlier onset of AMI (95% CI 4.48−9.97; p < 0.001), an increase in BMI of 1 kg/m^2^ was associated with 0.95 years earlier onset of AMI (95% CI 0.70−1.20; p < 0.001), an increase in LDL cholesterol of 1 mmol/L was associated with 2.17 years earlier onset of AMI (95% CI 0.67−3.66; p = 0.005), and for known diabetes, the result was not statistically significant (0.59 yrs; 95% CI −2.46 to 3.64; p = 0.704). Surprisingly, known hypertension was associated with 4.54 years later onset of AMI (95% CI 2.36−6.72; p < 0.001).

**Table 4:** Multiple regression associations of age at onset of AMI (years) with SES adjusting for traditional coronary risk factors and ancestry *BMI* Body mass index

	Model adjusting for traditional coronary risk factors			Model adjusting for ancestry			Model adjusting for traditional coronary risk factors and ancestry		
	Coef.	95% CI	p-value	Coef.	95% CI	p-value	Coef.	95% CI	p-value
High SES group	3.28	1.11 to 5.45	0.003	1.81	−0.55 to 4.17	0.132	0.80	−1.39 to 2.98	0.474
Stated active smoking	−7.22	−9.97 to −4.48	0.000				−7.00	−9.64 to −4.36	0.000
Diabetes	−0.59	−3.64 to 2.46	0.704				1.37	−1.63 to 4.36	0.369
Hypertension	4.54	2.36 to 6.72	0.000				3.93	1.84 to 6.03	0.000
BMI (1 kg/m^2^)	−0.95	−1.20 to −0.70	0.000				0.94	−1.18 to −0.69	0.000
LDL-cholesterol (1 mmol/L)	−2.17	−3.66 to −0.67	0.005				−1.89	−3.45 to −0.34	0.018
Use of statins	0.64	−1.92 to 3.20	0.624				1.01	−1.50 to 3.52	0.427
Presumed non-Northwest-European ancestry				−13.20	−16.63 to −9.77	0.000	−11.61	−14.79 to −8.43	0.000

*Coef.* coefficient, *CI* confidence interval

#### Ancestry

When adjusting for presumed ancestry, the difference in age at onset of AMI between the low SES and high SES group was no longer statistically significant (1.81 yrs; 95% CI −0.55 to 4.17; p = 0.132) (Table 4). Presumed non-Northwest-European ancestry was associated with 13.20 years earlier onset of AMI (95% CI 9.77−16.63; p < 0.001).

### Socioeconomic status

To assess the presence of individual characteristics of SES that are relevant to age at AMI onset, we performed a regression analysis adjusting for both presumed ancestry and traditional coronary risk factors. Then, the association between age at AMI onset and SES groups was further decreased (0.80 yrs; 95% CI −1.39 to 2.98; p = 0.474) (Table 4). Presumed non-Northwest-European ancestry was associated with an 11.61 years earlier onset of AMI (95% CI 8.43−14.79; p < 0.001), cigarette smoking with 7.00 years earlier onset of AMI (95% CI 4.36−9.64; p < 0.001), an increase in BMI of 1 kg/m^2^ by 0.94 years earlier onset of AMI (95% CI 0.69−1.18; p < 0.001), an increase in LDL cholesterol of 1 mmol/L by 1.89 years earlier onset of AMI (95% CI 0.34−3.45; p = 0.018), and for known diabetes, which was associated with 1.37 years later onset of AMI, the result was not statistically significant (95% CI −1.63 to 4.36; p = 0.369). Known hypertension was again associated with a protective effect, here constituting 3.93 years higher age at onset of AMI (95% CI 1.84−6.03; p < 0.001).

## Discussion

This study found that individuals with a low neighbourhood-level SES were 4.9 years younger at hospital admission with AMI and had a higher prevalence of past AMI than those with a high neighbourhood-level SES. When only comparing patients with a first time AMI, the difference increased to 6.1 years. The socioeconomically disadvantaged were also more likely to smoke and have diabetes. Traditional coronary risk factors could only account for part of the association between neighbourhood-level SES and age at AMI onset, whereas presumed ancestry could explain the entire association.

Our finding of an increased risk of AMI among individuals with a low SES is consistent with earlier evidence that socioeconomically disadvantaged are disfavoured in various aspects of CHD [6, 10, 11, 26]. However, some of these disparities might be attributable to socioeconomic differences in quality of care rather than other fundamental aspects of SES. For example, a Finnish registry study found that AMI patients with a low SES had a longer delay from AMI onset to medical presence, a lower likelihood of being admitted to a specialist hospital when residing in rural areas, and a lower likelihood of being prescribed the best available treatment and secondary prophylaxis when compared with those with a high SES [27]. In a recent systematic review, high SES populations were shown to consult specialised physicians more often than low SES populations, and the authors hypothesised that travel distance and patient preferences might be explanatory causes [28]. Similar findings have been demonstrated in Germany, where low SES has also been associated with less frequent use of prevention and health promotion services [29, 30].

A study from Bremen, Germany, which also used postal codes to categorise AMI patients in various socioeconomic groups and a myocardial infarction registry to identify patient characteristics, found an inverse association between SES and STEMI incidence and a poorer prognosis for the low SES group [31]. Although socioeconomic differences in door-to-balloon-time, PCI rates, or standard medication prescribed at hospital discharge were not found, socioeconomic differences in quality of care earlier in life may have contributed to the excess AMI incidence observed in the low SES population. The study population being drawn from a large region with multiple local hospitals may have facilitated this. Thus, an advantage of our study is that we included individuals from very nearby regions and that all individuals had the same local hospital. Nevertheless, our study population had among the largest differences in neighbourhood-level SES in Norway (Table 1). Another strength of our study is that SES can be expected to have less impact on the quality of care due to Norway’s universal healthcare system. All residents in Norway are covered by a national health security system with a universal tax-funded access to primary and secondary health care, including primary and secondary preventive drugs. Overall, we believe to have minimised bias due to different medical attention, treatment, and follow-up from the health and social services.

Our results demonstrate an approximately twice as high proportion of smokers and diabetic patients in the low SES group compared with the high SES group, also after individuals with presumed non-Northwest-European ancestry were excluded. This adds to previous research that shows an accumulation of traditional coronary risk factors in individuals with a low SES [10, 32, 33]. It should also be noted that low SES has been associated with a worse risk factor target achievement and less optimal secondary prevention after AMI [34]. For LDL cholesterol, BMI, and hypertension, we did not find statistically significant differences between the high and low SES regions. These findings are comparable with the results of Hamad et al., which demonstrated a great difference in the proportion of smokers and diabetics and a much smaller difference in mean LDL cholesterol, BMI, and blood pressure between individuals with a low and high SES [10]. A surprising result in our study was that patients in the low SES group with presumed Northwest-European ancestry and who were not on treatment with statins at the time of admission had a significantly lower value of LDL cholesterol than corresponding patients in the high SES group. Due to a presumed healthier lifestyle in regions with a high SES, we have no explanation for this observation. This may be due to play of chance, but may also partially be related to a more frequent intake of low fat and low cholesterol diet in the low SES population. Furthermore, we were surprised to find that individuals with known hypertension have a later onset of AMI. A partial explanation could be that persons with a previous diagnosis of hypertension are treated with medications like statins, β-blockers, and renin-angiotensin-system inhibitors, eliciting a primary prophylactic effect against AMI [35]. Furthermore, these patients may have a healthier diet and be more physically active, aiming to reduce blood pressure and protect from future disease.

Whether the main cause of the association between low SES and cardiovascular risk is due to individual characteristics of low SES or accumulation of traditional coronary risk factors in low SES individuals has been a subject of great debate. It is now widely accepted that traditional coronary risk factors do not fully explain social inequalities in CHD, and the estimates of the excess risk of low SES attributed to traditional coronary risk factors varies from 15 to 30% in the Whitehall study [14] to 40% in a recent study by Hamad et al. [10]. This study found that traditional coronary risk factors could only explain part of the association between neighbourhood-level SES and age at AMI onset, giving further support to this view. It should also be noted that others have argued that even though traditional coronary risk factors only have a modest role in explaining relative socioeconomic differences in CHD, they account for the majority of absolute socioeconomic differences in CHD as they account for most cases of CHD in the general population [13].

However, there has been less attention to whether ancestry may be of importance for explaining socioeconomic inequalities in CHD [10, 14, 15], even though ancestry has been shown to act in a complex interaction with SES and be a cardiovascular risk factor independent of SES [16, 17]. When studies have accounted for ancestry in their analyses, socioeconomic differences in CHD have usually persisted. For example, a US study found that living in a socioeconomically deprived neighbourhood was associated with a higher incidence of CHD in both black and white populations [36]. Furthermore, a meta-analysis with data from 48 independent prospective cohort studies found an inverse association between SES and cardiovascular disease (CVD) mortality after adjusting for ancestry and traditional coronary risk factors [37]. In view of prior studies, an interesting result of our study was that ancestry could explain the entire association between SES and age at AMI onset, suggesting that ancestry explain all socioeconomic inequality in risk of AMI. However, as we did not have data on individual-level SES or mean SES by ancestry in the city districts, we had no means to control for whether ancestry was a proxy variable for individual level SES in our study sample, and thus our results have to be interpreted with caution. Nevertheless, our study indicates that ancestry, along with traditional coronary risk factors and independent aspects of SES, has a role in mediating socioeconomic inequalities in CHD.

If our findings can be confirmed by future research, health measures to address socioeconomic inequality in CHD should also be directed at the characteristics of first-, second-, and maybe later-generation immigrants which are decisive for cardiovascular risk, as health measures directed only at SES and traditional coronary risk factors will probably have an insufficient effect. More research is needed to identify these characteristics, but at least four reasons why ancestry matters for health outcomes after accounting for SES have been identified [17]. First, adversity throughout life, such as poverty, abuse, and traumatic stress, has been proven to relate to ancestry and affect physical and mental health markers later in life, including cardiovascular function. Second, minorities at comparable SES as the majority population measured by a particular SES-indicator still often have a socioeconomic disadvantage measured by other common SES-indicators. Third, both intentional and unintentional ethnic discrimination may affect health. Fourth, minorities are more exposed to psychological stressors, such as discrimination, socioeconomic disadvantage, living in a foreign country away from friends and family, and adapting to a new language and culture. Furthermore, it might be argued that cultural and language barriers in this particularly vulnerable population can lead to less susceptibility to primary prevention and lower seek-out of health care and lower compliance of recommended lifestyle and prophylactic treatment. Other potential factors are comorbidities and genetics [38].

Regardless, measures to address socioeconomic disparities in CHD will also have to address socioeconomic inequalities and associated pathways linking SES to CHD. Psychosocial stressors, limited economic and educational opportunities, and social norms have been proposed mediating factors [10]. Allostatic load from toxic stresses of living with social disadvantage has been suggested to lead to atherogenic biological changes acting through stress hormones, endothelial dysfunction, metabolic disturbances, and inflammation [39], while limited economic and educational opportunities can lead to reduced access to health care and a less healthy lifestyle [10]. Further, peer influence from individuals in the same sociodemographic group might also influence lifestyle choices [40].

In summary, socioeconomic inequalities in CHD appear to be rooted in both traditional coronary risk factors and fundamental social conditions affecting health. These social conditions appear to be defined by both ancestry and SES and act through partially overlapping mechanisms. The internal significances of these factors and causal pathways leading to increased risk of CHD should be further investigated in future research so that adequate measures can be implemented.

## Limitations

There are several potential limitations in this study. Since there was no data in the Norwegian Myocardial Infarction Register on ancestry, this had to be estimated based on the patient’s name and information in the patient’s journal. This information is only an estimate of reality, as, for example, some people with ancestry from countries outside Northwestern Europe may have typical Northwest-European names.

The registration of traditional coronary risk factors has some uncertainties. If not previously documented in the electronic patient journal or informed by the referring general practitioner, we only had information provided by the patient regarding known diabetes and hypertension, previous AMI, and smoking status. This may have led to a biased distribution of which patients remembered or wanted to state these parameters. For LDL cholesterol, BMI, and to a lesser extent for smoking status, there were a lot of missing data. Multiple imputation was used to address these missing values. We assume that the data were missing at random since there are no apparent reasons why the measurements of height and weight, measurement of LDL cholesterol, and asking for smoking status were forgotten in some patients and not in others. However, we cannot completely rule out that smoking status becomes a little more uncertain due to language barriers in some of the patients.

The Norwegian Myocardial Infarction Register does not have an exhaustive register of traditional coronary risk factors. There were no data on physical activity, diet, alcohol intake, or psychosocial stress, which are also important risk factors for AMI [41]. Due to missing these possibly confounding variables in our analyses when adjusting for traditional coronary risk factors, estimates of the significance of traditional coronary risk factors as a whole are likely to be underestimated. This may have resulted in the internal significance of the variables cigarette smoking, diabetes, hypertension, LDL cholesterol, and BMI being overestimated. The significance of presumed ancestry may also be overestimated since it may be the missing traditional coronary risk factors that explain our findings.

Another aspect of our data is that we included only patients with NSTEMI. Therefore, we do not have the basis for commenting on patients with STEMI. However, since the pathophysiological mechanisms behind the development of STEMI and NSTEMI are the same, we believe that our results also apply to patients with STEMI. There may also be a bias in the selection of patients with NSTEMI sent to Diakonhjemmet Hospital for treatment since Diakonhjemmet Hospital only received 41.22% of all NSTEMI patients from the north-eastern districts of Oslo during the relevant period.

We had no methods to measure individual-level SES, but previous research has shown that neighbourhood measurements work as good as individual measurements for estimating subjects’ SES [42].

## Conclusion

Our study supports previous research showing increased risk of AMI and increased prevalence of traditional coronary risk factors in individuals with a neighbourhood-level socioeconomic disadvantage. Our findings indicate that ancestry might have a role in explaining the increased risk, but future research is needed to confirm this result and to identify the characteristics of individuals of another ancestry than the majority population which are of importance to cardiovascular risk, so that adequate societal measures can be implemented.

## Data Availability

An anonymised version of the dataset generated and analysed during the current study are available in the Harvard Dataverse repository, 10.7910/DVN/5X0MNC [43]. Raw data from the Norwegian Myocardial Infarction Register, that was used to generate variables on the patients’ assumed ancestry and city-district group, is not made available as it contains personal data, but can be applied for at helsedata.no.

## Abbreviations

AMI: Acute myocardial infarction.
BMI: Body mass index.
CHD: Coronary heart disease.
CI: Confidence interval.
CVD: Cardiovascular disease.
EU: European Union.
LDL: Low-density lipoprotein.
NOK: Norwegian kroner.
NSTEMI: Non-ST-elevation myocardial infarction.
SES: Socioeconomic status.
Yrs: Years.
