# Correction: Impact of neighbourhood-level socioeconomic status, traditional coronary risk factors, and ancestry on age at myocardial infarction onset: A population-based register study

**DOI:** 10.1186/s12872-022-02938-6

**Published:** 2022-11-23

**Authors:** Mathias Øie Kolden, Ståle H. Nymo, Erik Øie

**Affiliations:** Department of Internal Medicine, Diakonhjemmet Hospital, Oslo, Norway

**Correction: BMC Cardiovasc Disord 22, 447 (2022)**

**https://doi.org/10.1186/s12872-022-02880-7**

Following publication of the original article [1], in Table 2, the footnote has been placed with the figure cation. So, the table should read as follows:

**Table 2:** Number of individuals with missing data for all variables in the western (mean high SES) and north-eastern (mean low SES) districts of Oslo

	Total population (*N*=606)		Presumed Northwest-European ancestry (*N*=531)	
	Western districts (*N*=391)	North-eastern districts (*N*=215)	Western districts (*N*=375)	North-eastern districts (*N*=156)
Smoking status	73	27	69	22
Hypertension	29	1	27	1
Diabetes mellitus	23	2	22	2
LDL-cholesterol	180	98	173	75
BMI	151	100	143	66
Use of statins	23	2	22	2
Previous AMI	30	4	29	4

*BMI* Body mass index

The original article has been corrected.
